# Sojourn of a Cherry Stone Nine Months in the Air Passages

**Published:** 1845-06

**Authors:** 


*Sojourn of a Cherry stone nine months in the Air Passages.* By M. Maslieurat-Lagemard.—A woman, 54 years of age, without cause known to herself, was seized with fits of coughing, which came on every eight or nine days, and continued for several minutes. She had no feverish symptoms, nor pain of chest, nor unusual respiratory murmur. As the cough was always attended with sensation of suffocation, she asked medical advice; but as no cause could be detected to account for the cough, it was deemed nervous, and treated accordingly, but without affording any relief. Nine months after its appearance, during a fit of coughing, she expectorated a hard body, which on examination proved to be a cherry-stone surrounded with a calcareous incrustation.—*Ibid, from Gazette Medicale de Paris.*

